# A Facile Approach for Preparing Ag Functionalized Nonwoven Polypropylene Membrane to Improve Its Electrical Conductivity and Electromagnetic Shielding Performance

**DOI:** 10.3390/ma12020296

**Published:** 2019-01-18

**Authors:** Dongfeng Shao, Hongwei Zhang, Lizhen Tao, Kan Cao, Qufu Wei

**Affiliations:** 1 Changzhou Vocational Institute of Textile and Garment, Changzhou 213164, China; weihongz@126.com (H.Z.); taolizhen98@126.com (L.T.); 13775126362@139.com (K.C.); 2 Changzhou Key Laboratory of eco Textile Technology, Changzhou 213164, China; 3 Key Laboratory of Eco-Textiles, Ministry of Education, Jiangnan University, Wuxi 214122, China

**Keywords:** polypropylene, Ag, electrical conductivity, electromagnetic shielding

## Abstract

The commonly used preparation methods of polypropylene functionalization require special equipment to be put into use or take a long time, which limits its application. Therefore, a simple and economical method for preparing silver functionalized nonwoven polypropylene membrane was studied herein. Triethanolamine was first coated on the surface of the polypropylene, and then Ag was deposited on the surface of polypropylene using a continuous reduction reaction of triethanolamine and silver ions. Surface morphology, crystal structure, and surface chemistry during the preparation of Ag functionalized nonwoven polypropylene were investigated. The electrical conductivity, electromagnetic shielding properties, and washing durability of the treated nonwoven polypropylene were also studied. It was found that Ag was uniformly deposited on the surface of the nonwoven polypropylene, and the coating reaction did not change the chemical structure of the polypropylene. The crystallinity and thermal stability of polypropylene were improved after silver coated polypropylene. The washing experiment results showed that the weight gain rate of the treated nonwoven relative to the untreated sample after the 90 min washing ranged from 6.72% to 9.64%. The resistance test results showed that the maximum surface resistivity of Ag coated nonwoven polypropylene was about 1.95 × 10^5^ Ω, which was 64,615 times lower than the original. In addition, the results showed that the maximum electromagnetic shielding effectiveness of the Ag coated nonwoven polypropylene was about 71.6 dB, showing a very good electromagnetic shielding effect.

## 1. Introduction

In recent years, with the rapid improvement of electronic technology and artificial intelligence, more and more electronic products are being used, and electromagnetic radiation generated by electronic products has caused widespread concern, which resulted in an increase in demand for conductive textiles and electromagnetic shielding textiles. Different methods have been used to improve the electrical conductivity and electromagnetic radiation resistance of textiles, such as embedded conductive fibers [1] or conductive yarns [2] in fabrics, and coated conductive layers on the surface of fabrics [3]. These methods improved the electrical conductivity of textiles while achieving electromagnetic shielding of textiles by absorbing or reflecting electromagnetic waves [4].

In the research of conductive and electromagnetic shielding textiles, metallic silver is one of the most widely used materials for conductive and electromagnetic protective products because of its excellent conductive properties, electromagnetic wave reflection properties, and easy preparation of conductive fibers, conductive yarns, or conductive coatings. For example, surface-modified and electroless silver-plated acrylonitrile fabrics achieved a good electromagnetic shielding performance of 40–80 dB [5]. M.S. Ozenet et al. [6] reported that the shielding effect of nonwoven made of 6.7 dtex and 3.3 dtex conductive Ag/PA66 staple fibers was about 69–80 dB when the weight of nonwoven was only 0.04 g/cm^2^.

Nonwoven polypropylene is widely used in textiles, packaging, medical supplies, and filter materials due to their resistance to acid and alkali corrosion, good strength, low cost, and convenient processing [7]. However, due to the lack of conductive groups in its molecular structure, the conductivity is poor, and the static electricity generated by friction during use is not easily dissipated, which affected its use. Therefore, silver functionalized polypropylene may be a good choice, not only to improve the conductivity and electromagnetic protection of polypropylene, but also to enhance its antibacterial properties.

Different technologies have been used for silver functionalized polypropylene, such as preparation of polypropylene/silver composite fibers [8], magnetron sputtering deposition of silver on polypropylene surface [9], silver deposition on polypropylene surface after plasma modification [10], silver deposition on polypropylene surface after photografting [11], and silver deposition on polypropylene surface after gamma radiation-induced grafting [12]. Among them, preparation of polypropylene/silver composite fiber required preparation of silver nanoparticles, polypropylene grafting, or surface modification of silver nanoparticles, and a blending spinning process. Although the polypropylene/silver composite fiber has better antibacterial property and antistatic property, more preparation steps are required, and the discontinuous distribution of the silver particles in the fiber is not beneficial for improving the electromagnetic shielding performance. The magnetron sputtering method is simple but requires equipment investment. Other methods require surface-modified polypropylene and then silver deposition, which has many steps and equipment investment is also required. Therefore, silver functionalized polypropylene technology with simple operation and low cost should be studied to meet the needs of industrial development.

From the point of view of the functionalization of the fabric, the application of silver to the surface of the fiber or fabric is most advantageous for conductivity and electromagnetic shielding. Although silver ions can be easily reduced to silver, due to the lack of reactive groups on the polypropylene fiber surface, it is difficult for the polypropylene to adsorb silver ions to the surface of the fibers and to reduce the deposition of silver to the surface. Therefore, adsorbing silver ions to the surface without changing the chemical structure of the polypropylene surface is the key to solving the problem. Fortunately, Hajar Poortavasoly et al. [13] reported a method of simultaneously modifying the surface of polyester with triethanolamine and depositing silver onto the surface of polyester. The triethanolamine used in this study provides a solution to the problem of adsorption and reduction of silver on the surface of polypropylene due to its adhesion, hydrophilicity, and reducibility. The innovative idea is to first a layer of triethanolamine is adhered on the surface of the polypropylene fiber. The silver ions are then adsorbed by the triethanolamine coated nonwoven polypropylene in solution, and reduced to silver seeds between the fiber surface. With the continuous reaction of reductant and silver ions in solution, more silver ions are reduced and grow on the silver seed on the surface of the fibers. Finally, a metallic silver coating is formed on the surface of the fabrics.

In this study, Ag functionalized nonwoven polypropylene was prepared using this facile approach. The morphology, crystalline state, chemical structure, and properties of Ag functionalized nonwoven polypropylene were characterized. The functional membrane exhibited excellent resistivity, which is beneficial to eliminate static electricity generated by friction and its potential application in smart textiles. In addition, the functional membrane demonstrated its practical application potential by detecting satisfactory electromagnetic shielding effectiveness. In short, this work provides a simple, convenient, and effective method for preparing a Ag functionalized nonwoven polypropylene membrane.

## 2. Materials and Methods

### 2.1. Materials

Spunbond-meltblown-spunbond nonwoven polypropylene (36 g/m^2^) was purchased in the market. The diameter of spunbonded fiber ranges from 1.65 µm to 1.83 µm, and the average diameter was about 1.74 µm. Meltblown fibers had an average diameter of 290 nm ranging from 130 nm to 640 nm. The thickness of the sample was about 0.23 mm. The nonwoven polypropylene was repeatedly washed with ethanol and water to remove the adherend prior to use. Silver nitrate (AgNO_3_) and triethanolamine (TEA) were purchased from Shanghai Chemical Reagent Co., Shanghai, China.

### 2.2. Preparation of Ag Coated Nonwoven Polypropylene

**Figure 1:**
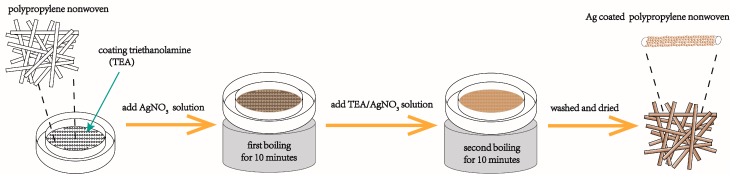
Schematic diagram of preparing Ag functionalized nonwoven polypropylene.

Figure 1 shows the preparation process of Ag functionalized nonwoven polypropylene. First, a nonwoven polypropylene was mounted on a circular bamboo sample holder having a diameter of 16 cm with the smooth side of the sample facing down. Then, 20 mL of triethanolamine was added to a 20 cm diameter crystallizing dish. Next, the sample holder was placed horizontally in a crystallizing dish and the sample was squeezed with a spoon to ensure that the polypropylene film was sufficiently wetted and wrapped with triethanolamine. Then, 0.4 g of silver nitrate was dissolved in 200 mL of water, and then the solution was added to the crystallizing dish. Next, the crystallizing dish was placed on an electric furnace and heated to boiling for 10 min. Meanwhile, 0.4–0.8 g of silver nitrate and 20–60 mL of triethanolamine were dissolved in 170 mL of distilled water. Then, the prepared silver nitrate/triethanolamine solution was slowly added to the crystallizing dish. Next, the crystallizing dish was heated to boiling for 10 min again. Finally, the treated sample was repeatedly washed with distilled water to remove the surface adsorbed reagent, and then dried at 60 °C. The preparation conditions of the sample are shown in Table 1.

### 2.3. Characterizations

Surface morphological changes during Ag functionalized propylene nonwoven were examined by scanning electron microscopy (SEM, S-4800, Hitachi, Tokyo, Japan). Magnification of SEM was 500 and 30,000, respectively.

The crystal state of the nonwoven polypropylene before and after the Ag coating was tested using an X-ray diffractometer (XRD, D/max 2500 PC, Rigaku, Tokyo, Japan) with Cu Kα radiation (I = 1.54056). The crystallinity of polypropylene was analyzed using MDI Jade 6.0 (Livermore, CA, USA) and the calculation formula was as follows [14]:

(1) $$X=\frac{\sum I_{c}}{\sum I_{c}+\sum I_{a}}\times 100\%$$

where $\sum I_{c}$ is the integral intensity of total polycrystal diffraction of crystalline portion and $\sum I_{a}$ is the scattering integral intensity of amorphous portion.

Fourier transform infrared spectroscopy (FT-IR, IS50, ThermoFisher, Waltham, MA, USA) with a range of 4000–400 cm^−1^ and a resolution of 4 cm^−1^ was used to analyze the chemical changes of nonwoven polypropylene during Ag functionalization. X-ray photoelectron spectroscopy (XPS, Escalab 250Xi, ThermoFisher, Waltham, MA, USA) with monochromatic Al Kα radiation (hν = 1486.7 eV) was used to analyze the surface element content and elemental valence of the Ag functionalized nonwoven polypropylene.

Thermal stability of silver functionalized polypropylene before and after treatment was measured by thermogravimetric analysis (TG, 209 F3, Netzsch, Selb, Germany) in a nitrogen atmosphere. The temperature of the test ranged from 50 to 600 °C, and the heating rate was 10 °C/min.

### 2.4. Weight Gain Rate and Washing Stability

Weight change before and after silver coated nonwoven polypropylene was calculated using the weight gain rate, where the weight gain rate was determined as follows:

(2) $$\Delta W_{1}=\frac{\left(W_{2}-W_{1}\right)}{W_{1}}\times 100\%$$

where Δ*W*_1_ is the weight gain rate (%), *W*_1_ are the weights of the samples before treatment (g), and *W*_2_ are the weights of the samples after treatment (g).

The washing stability of the Ag-coated nonwoven polypropylene was evaluated using the weight change rate of the samples before and after washing in the domestic washing machine for 30, 60, and 90 min. The washing process is that 10 g samples was washed at 30 °C in 4 L water for 30 min, and 5 g standard detergents was also added into the water during the washing process. The standard detergent used in this study is the ECE (ECE refers to the European Colorfastness Establishment) reference detergent 98 without optical brightener according to ISO 6330. The sample was then washed with 2 L water for 2 min. Finally, the sample was taken out and dried. The weight loss rate before and after washing is used to evaluate the washing stability, and the calculation is as follows:

(3) $$\Delta W_{2}=\frac{\left(W_{3}-W_{4}\right)}{W_{3}}\times 100\%$$

where Δ*W*_2_ is the weight loss rate (%), *W*_3_ are the weights of the unwashed samples (g), and *W*_4_ are the weights of the washed samples (g).

### 2.5. Surface Resistance and Electromagnetic Shielding

The surface resistivity measurement of before and after Ag coated nonwoven polypropylene specimens was carried out in accordance with EN 1149-1 standard, using a textile electrostatic resistance tester (FJD-D, Yuanfeng, Xian, China). The fabric sample was cut into a circular shape with a diameter of 13 cm. The surface resistance of the fabric was tested at 20 °C and 65% relative humidity. The voltage of 100 V was applied to the fabric, and its resistance was tested after 15 s. If the resistance was less than 10^5^ Ω, the test voltage was adjusted to 50 V. Four different locations were tested for each sample and the average was calculated. The resistivity was calculated as follows:

(4) ρ=k×R

where *ρ* is the calculated surface resistivity (Ω); *R* is the measured resistance (Ω); and *k* is the geometrical factor of the electrode, where for this electrode, the factor is 19.8.

The electromagnetic shielding effectiveness of the Ag coated nonwoven polypropylene was investigated by shielding effectiveness coaxial fixture (R913E, Darong, Wenzhou, China) and a vector network analyzer (DEVISER NA7300A, Deli, Tianjin, China) with the frequency ranging from 150 MHz to 3 GHz according to the ASTM-D4935-2010 standard at room temperature. The fabric sample was cut into a circular shape with a diameter of 13 cm and then placed between the flanges for measurement. Then, the electromagnetic shielding effectiveness was recorded.

## 3. Results and Discussion

### 3.1. Crystalline Structure Analysis

**Figure 2:**
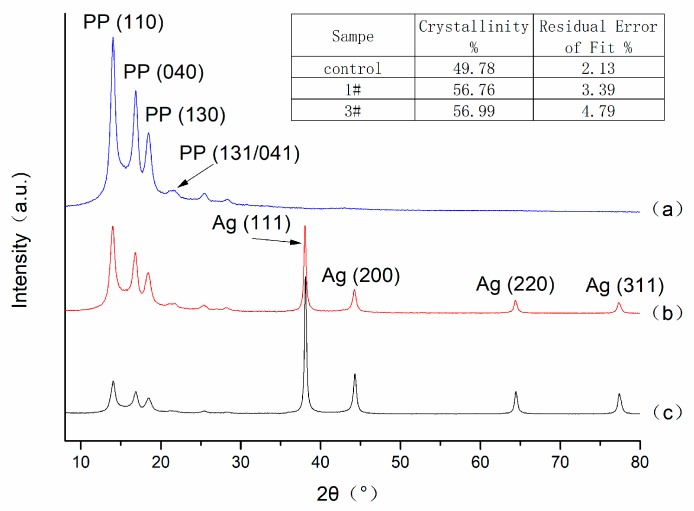
X-ray diffractometer (XRD) patterns of (**a**) nonwoven polypropylene, (**b**) Ag coated nonwoven polypropylene (1#), and (**c**) Ag coated nonwoven polypropylene (3#).

The X-ray diffraction (XRD) patterns of nonwoven polypropylene, 1# sample and 3# sample are illustrated in Figure 2. From Figure 2a, it can be observed that there are reflection peaks at 2θ = 14.0°, 16.9°, 18.4°, and 21.8°, which corresponded (100), (040), (130), and (131/041) planes of polypropylene [15], respectively. After the first half of the Ag coating process, it can be seen from Figure 2b that more peaks are detected in the XRD curve at 2θ = 14.0°, 16.9°, 18.4°, 21.8°, 38.0°, 44.2°, 64.4°, and 77.4°. Except for the diffraction peaks of nonwoven polypropylene, such as 2θ = 14.0°, 16.9°, 18.4°, and 21.8°, the other peaks at 2θ = 38.0°, 44.2°, 64.4°, and 77.4° correspond to the (110), (200), (220), and (311) crystal plane of Ag [16]. These results indicate that the first half of the Ag coating process had deposited silver on the surface of the nonwoven polypropylene. From Figure 2c, it can be observed that the diffraction peaks of Ag at 2θ = 38.0°, 44.2°, 64.4°, and 77.4° increased. This was because the 3# sample was obtained from continuous growing Ag on the 1# sample, which resulted in more Ag being reduced and deposited on the 3# sample to increase the grain size of the silver. In addition, it can be seen from the calculation results of crystallinity on Figure 2 that the crystallinity of the control sample, 1# sample and 3# sample were 49.78%, 56.76%, and 56.99%, respectively. This indicates that the silver functionalized nonwoven polypropylene process resulted in a slight increase in the crystallinity of the polypropylene, which may be due to the fact that the nonwoven polypropylene fixed to the circular bamboo sample holder was subjected to stretch deformation and molecular rearrangement under the impact of the boiling liquid.

### 3.2. Surface Morphology Analysis

**Figure 3:**
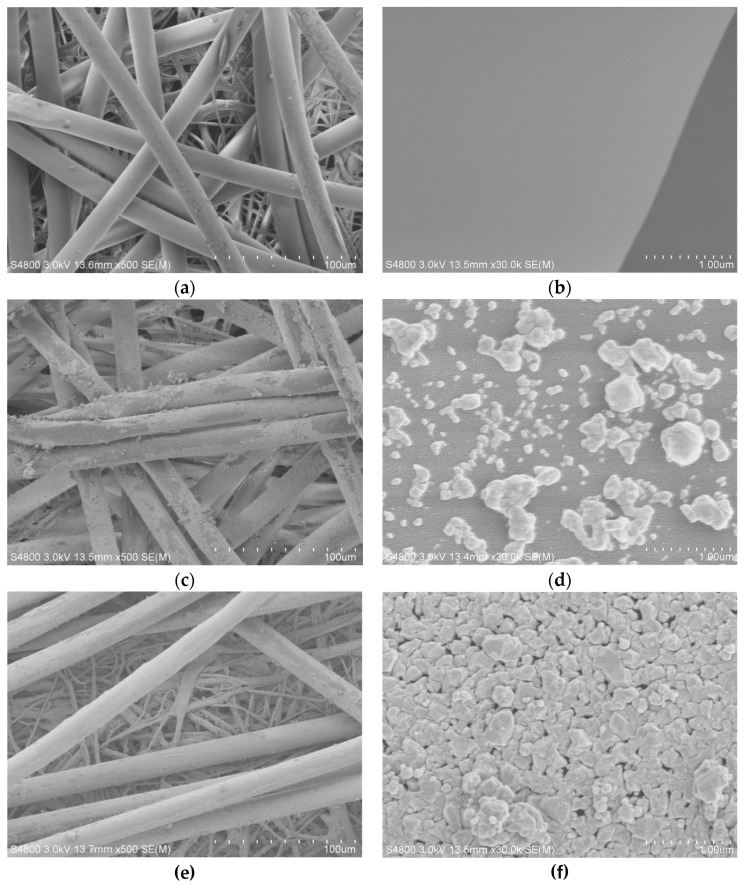
Scanning electron microscopy (SEM) images of (**a**,**b**)nonwoven polypropylene, (**c**,**d**) treated nonwoven polypropylene (1#), and (**e**,**f**) treated nonwoven polypropylene (3#).

The SEM images of treated and untreated nonwoven polypropylene are shown in Figure 3. From Figure 3a,b, it can be seen that the nonwoven polypropylene upper spunbond fiber surface and the lower meltblown fiber were relatively smooth. The presence of particles in the surface of the upper spunbond fibers in Figure 2a was due to gold coating during the preparation of samples for SEM testing. Compared with untreated nonwoven polypropylene, it can be observed that the surface of the nonwoven polypropylene (1#) showed significant changes after Ag coating, as is illustrated in Figure 3c,d. It can be observed that the surface of the fiber became rough and most of the surface was covered by particles, and the wrapping of these particles was uneven, as is shown in Figure 3c. This unevenness was due to the fact that triethanolamine coated on the surface of the fiber was washed by a water stream during the boiling reaction, resulting in too low of a concentration of triethanolamine on the surface of the fiber portion, such that these portions of the fiber surface were less likely to adsorb Ag ions and deposit Ag. In addition, there was particle agglomeration on the surface of the fiber because the particles in the solution were adsorbed. After magnifying 30,000 times, it can be seen that the particles deposited on the surface of the fiber were uniformly distributed and irregular in shape, ranging from 44 to 998 nm, as shown in Figure 3d. Figure 3e,f are SEM images of Ag-coated nonwoven polypropylene (3#), which was obtained after further growth of Ag on sample 1#. Compared with Figure 3c, it can be seen from Figure 3e that the surface of the meltblown fibers of the nonwoven polypropylene substrate and the surface of the spunbond fibers of the upper layer were covered by more uniform Ag particles. It can be further seen from Figure 3f that the particles were arranged neatly and densely on the surface of the fiber to form a thick coating on the surface of the fiber at a magnification of 30,000 times. These results indicate that Ag can be deposited well onto the surface of polypropylene fibers using this method.

### 3.3. Surface Chemistry Analysis

**Figure 4:**
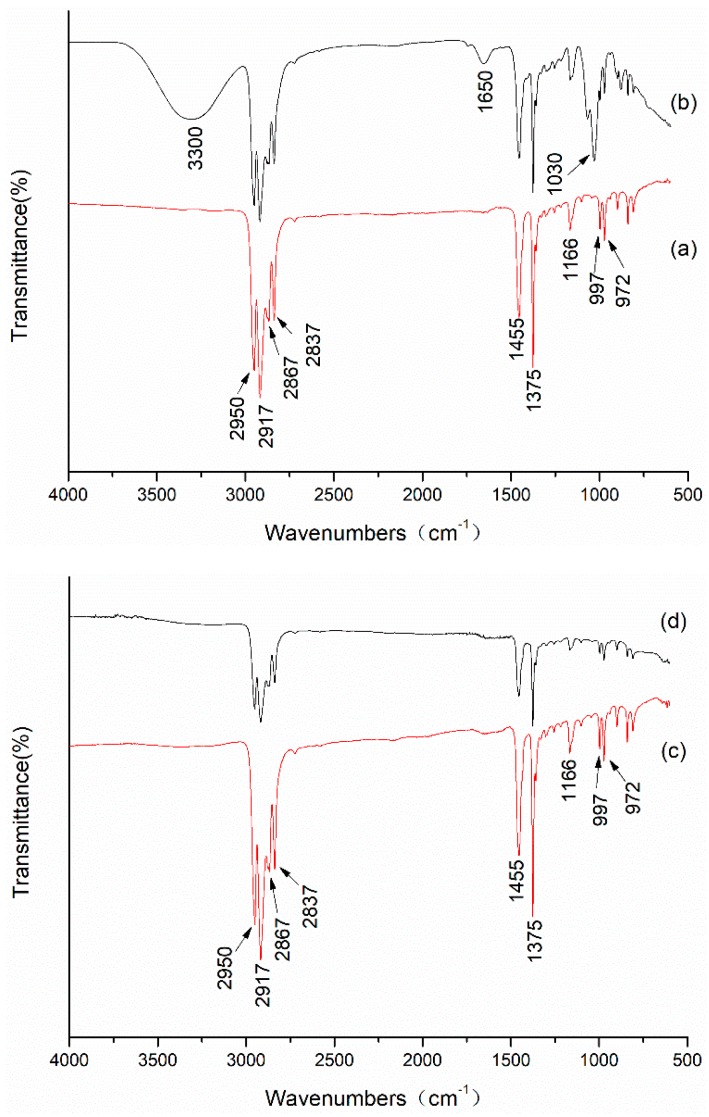
Fourier transform infrared spectroscopy ATR spectra of (**a**) untreated nonwoven polypropylene, (**b**) triethanolamine coated nonwoven polypropylene, (**c**) Ag coated nonwoven polypropylene (1#), and (**d**) Ag coated nonwoven polypropylene (3#).

Figure 4a shows the FT-IR/ATR spectra of untreated nonwoven polypropylene. As is shown in Figure 4a, the band at 2951 cm^−1^ and 2917 cm^−1^ were attributed to the asymmetric stretching vibration of CH_3_ and CH_2_ groups [17]. The CH_3_ and CH_2_ symmetric stretching were observed at the bands 2868 cm^−1^ and 2838 cm^−1^. The bands at 1455 cm^−1^ and 1375 cm^−1^ represent the symmetric and asymmetric scissoring vibrations of the methyl group. The bands at 1167 cm^−1^, 997 cm^−1^, and 972 cm^−1^ were due to the isotactic portion [18]. After coating polypropylene with triethanolamine, it can be seen from the infrared spectrum in Figure 4b that three new peaks appeared at 3300 cm^−1^, 1650 cm^−1^, and 1030 cm^−1^, besides the band of polypropylene. These three peaks were all caused by triethanolamine, wherein the band at 3300 cm^−1^ was due to the stretching vibration of the hydroxyl group of triethanolamine [19], the band at 1650 cm^−1^ was derived from the molecular bending O–H vibrations [20,21], and the band at 1030 cm^−1^ was caused by C–O bond vibration of triethanolamine [21]. Compared to untreated polypropylene, it can be seen from Figure 4c,d that the infrared spectrum of the Ag-coated polypropylene has no significant change. Moreover, there were no characteristic peaks, such as O–H and C–O of triethanolamine, in the infrared spectrum, which indicates that the method of depositing Ag on the surface of the polypropylene did not change the molecular structure of the polypropylene.

**Figure 5:**
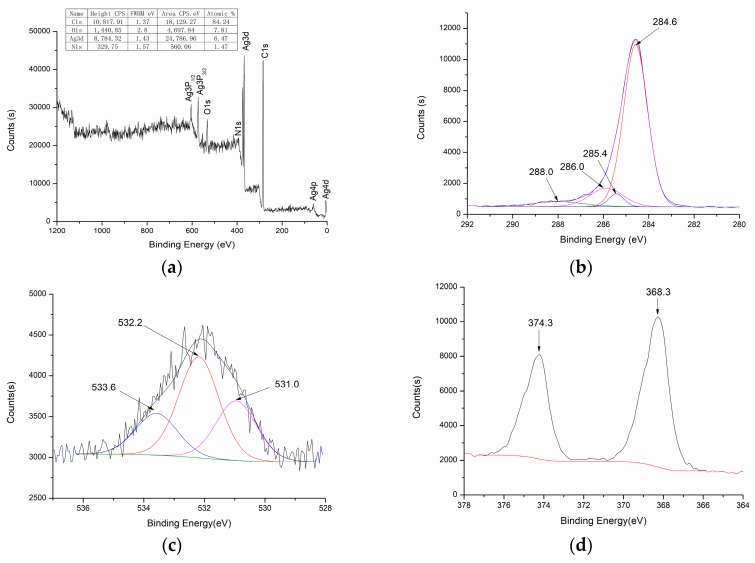
X-ray photoelectron spectroscopy of Ag coated nonwoven polypropylene (1#): (**a**) full spectrum, (**b**) C1s peak, (**c**) O1s peak, and (**d**) Ag3d peak.

X-ray photoelectron spectroscopy of Ag coated nonwoven polypropylene (1# sample) is shown in Figure 5a. C, Ag, O, and N elements are found on the surface of Ag-coated nonwoven polypropylene, as is shown in Figure 5a. The atomic contents of the elements in C1s, Ag3d, O1s, and N1s are 84.24%, 6.67%, 7.81%, and 1.47%, respectively. The high resolution XPS spectra of C1s, O1s, and Ag3d are shown in Figure 5b–d, respectively. As is shown in Figure 5b, the C1s spectrum of Ag coated nonwoven polypropylene contained four well-separated peaks at 284.6 eV, 285.4 eV, 286.0 eV, and 288.0 eV. The peaks at 284.6 eV, 286.0 eV, and 288.0 eV were associated with C–H/C–C, C–OH, and C=OH [22,23], respectively. The peak at 285.4 eV was related to C–N structure [24]. These results indicate that there is a residue remaining on the surface of the fiber after the reduction reaction of triethanolamine with silver ions on the surface of the fiber. From Figure 5c, it can be observed that the O1s spectrum was fitted to three peaks of 531.0 eV, 532.2 eV, and 533.6 eV. The peak at 531.0 eV and 532.2 eV were associated with C=OH and C–OH, respectively [25]. The binding energy of C=O was only 531.0 eV because the transfer of charge from the Ag atom to the O=C functional group leads to a decrease in binding energy. The peak at 533.6 eV was attributed to surface absorbed hydroxyl oxygen [26]. In addition, it can be seen from the energy spectrum O1s that no peak can be fitted at a binding energy of less than 530 eV. Because the peak of the oxygen binding energy of AgO and Ag_2_O was between 528 eV and 530 eV [27], this reveals that there is no AgO or Ag_2_O on the fabric surface. Which could be attributed to the residual C=OH and C–OH on the fabric surface protecting silver from oxidation. From Figure 5d, it can be seen that the two peaks of Ag3d were centered at 368.3 eV and 374.3 eV. The difference in binding energy between the two peaks was 6.0 eV, indicating that elemental silver was deposited on the surface of the fiber [28].

**Figure 6:**
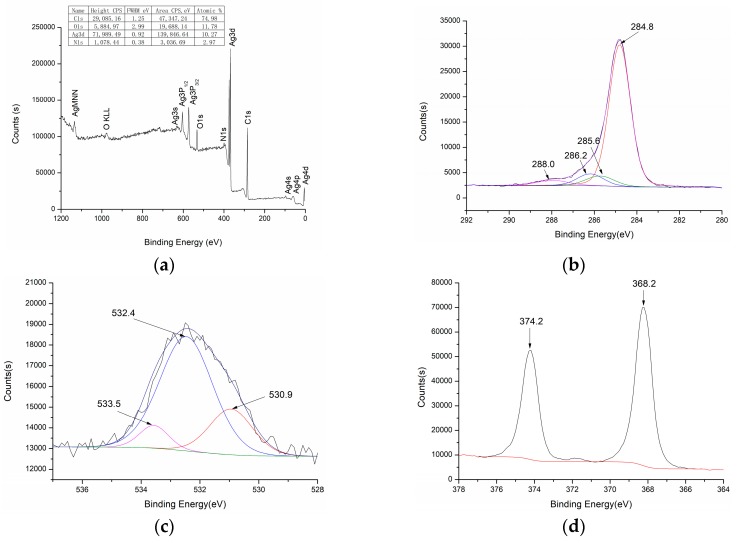
X-ray photoelectron spectroscopy of Ag coated nonwoven polypropylene (3#): (**a**) full spectrum, (**b**) C1s peak, (**c**) O1s peak, and (**d**) Ag3d peak.

Compared with the 1# sample, the XPS pattern of the silver functionalized nonwoven polypropylene (3#) did not change significantly, as shown in Figure 6. The surface of the 3# sample was still composed of C, O, Ag, and N elements, but the content of O, Ag, and N elements on the surface of 3# sample was slightly higher than that of 1# sample. Similarly, the structures C–H/C–C, C–OH, C=OH, and C–N in Figure 6b and the structures C=OH, C–OH, and C=O in Figure 6c were also observed in the surface of Ag functionalized propylene nonwoven. The binding energy of 368.2 eV and 374.2 eV corresponded to Ag 3d_5/2_ and Ag 3d_3/2_ further proves that elemental silver was deposited on the surface of the propylene nonwoven by this method.

### 3.4. Thermogravimetric Analyses

**Figure 7:**
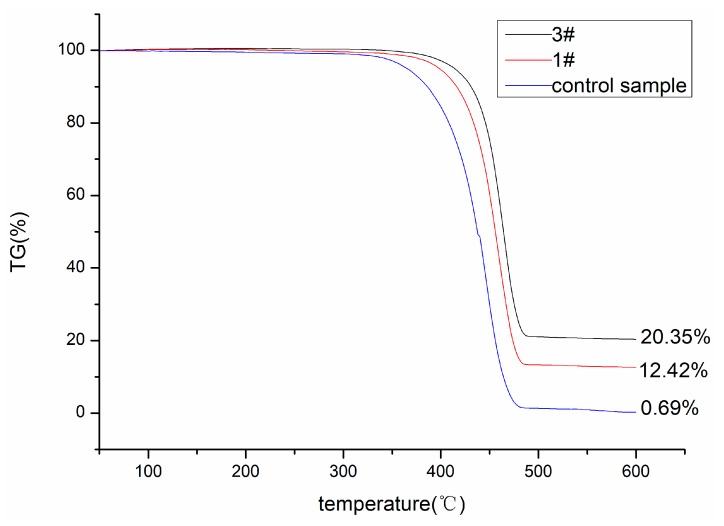
TG curves of control and Ag coated nonwoven polypropylene.

The thermogravimetric curves of the samples before and after silver functionalized nonwoven polypropylene are shown in Figure 7. As can be seen from Figure 7, the thermal decomposition curves of the control sample and the Ag functionalized polypropylene (1# and 3#) were similar. However, the control sample weight loss was concentrated in the range of 312 °C to 478 °C, the thermal weight loss of the 1# sample was mainly ranges from 335 °C to 489 °C, and the thermal weight loss of the 3# sample mainly ranged from 345 °C to 493 °C. These results indicate that the silver coated on the surface of the polypropylene fibers may have formed a physical protective barrier [29] on the surface of the polypropylene fibers. It can also be observed that the remaining weight of the control sample, 1# sample, and 3# sample was 0.69%, 12.58%, and 20.36% of the original weight at 600 °C, respectively. This was mainly because the amounts of Ag deposited on the surface of the nonwoven polypropylene were different. Silver was not easily decomposed at 600 °C, so the more silver that was coated on the surface of the nonwoven, the greater the weight of the remainder at this time.

### 3.5. Weight Gain Rate and Washing Stability

The weight change before and after Ag coated polypropylene is shown in Table 2. From Table 2, it can be seen that the weight increase rate of the Ag coated nonwoven polypropylene (1#) prepared in the first half of the Ag coating process was about 8.78%, and the weight increase rate of the Ag coated nonwoven polypropylene (2#, 3#, 4#, 5#, 6#) prepared using the whole Ag coating process ranged from 20.74% to 30.40%. The amount of Ag deposited on the surface of polypropylene in the complete preparation process was much larger than the amount of silver deposited on the surface of the polypropylene in the first half of the process. This was because the first half of the Ag coating process coated a layer of Ag on the surface of the fiber, which made it easier to absorb Ag ions, reducing and depositing Ag on the fiber surface. Compared with the samples of 2#, 3#, and 4#, it can be seen that the weight of Ag deposited on the surface of polypropylene increased gradually with the increase of silver nitrate dosage from 0.4 g to 0.8 g in the second stage of the Ag coating process, which was attributed to the increase in the amount of silver nitrate so as to cause more Ag ions to be reduced and deposited on the surface of the fiber. Similarly, comparing the 3#, 5#, and 6# samples, it can be observed that in the second stage of silver coating, as the amount of triethanolamine was increased from 20 mL to 60 mL, the weight of silver deposited on the surface of the polypropylene was also increased. The reason may be that with the increase of the amount of triethanolamine in the coating reaction, the amount of triethanolamine attached to the polypropylene surface also increased, resulting in more Ag ions being adsorbed and reduced on the surface of the nonwoven polypropylene.

The weight change of the Ag coated nonwoven polypropylene after washing for 30 min, 60 min, and 90 min is shown in Table 3. It can be seen from Table 3, with the increase of washing time, the weight loss rate of the sample decreased. According to the data in the Table 3, the weight loss rate decreased with the increase of washing time. However, taking 3# as an example, after washing for 90 min, the weight was still 1.0161 g. Compared with untreated 3# sample’s weight of 0.9288 g in Table 2, the weight was still increased by 0.0873 g, and the weight gain rate was about 9.40%. This indicated that there were still many Ag particles coated on the surface of the fabric after 90 min of washing. The main reason for this phenomenon is that a large amount of Ag particles adsorbed on the nonwoven surface at the beginning of the washing stage were easily washed away because of the weak interaction with nonwoven polypropylene, so the weight loss rate was high. In addition, during the boiling process, although the thermal motion of the polypropylene molecule was intensified, the nonwoven polypropylene could not shrink because it was fixed by the circular bamboo sample holder, and would be stretched and deformed via the boiling liquid impact instead. The microcrystalline silver particles synthesized on the surface may have been embedded on the surface of the fiber under the continuous action of the boiling liquid. During the cooling process, the morphology of the polypropylene was solidified due to recrystallization. Also, it may be due to the structure of spunbond-meltblown-spunbond nonwoven that the outer spunbond fibers protected the silver coating on the inner meltblown fibers, making it difficult for them to fall off during washing. Therefore, the weight loss rate gradually decreased in the subsequent washing process, and a large amount of silver particles were deposited on the surface of the fiber after washing for 90 min.

### 3.6. Surface Resistance

Table 4 shows the surface resistance and resistivity of untreated nonwoven polypropylene and treated nonwoven polypropylene. Comparing with the control sample, it can be seen from Table 4 that the surface resistivity of Ag coated nonwoven polypropylene (1#) prepared by the first half of the Ag coating process decreased from 1.26 × 10^10^ Ω to 1.09 × 10^7^ Ω, a drop of about 1155 times. However, the maximum surface resistivity of Ag coated nonwoven polypropylene (2#, 3#, 4#, 5#, and 6#) prepared by the complete Ag coating process was about 1.95 × 10^5^ Ω, which is a reduction of about 64,615 times compared to the untreated samples. This indicates that this method of coating Ag on the surface of a nonwoven polypropylene can significantly improve its electrical conductivity. Comparing 2#, 3#, 4#, 5#, and 6# samples, it can be observed that the resistivity between these samples ranged from 1.82 × 10^5^ Ω to 1.95 × 10^5^ Ω, the difference was not very big, which was because Ag particles covered most of the surface of the fabrics and formed a stable conductive layer when Ag-coated nonwoven polypropylene were prepared under these conditions. These changes in the amount of silver nitrate and the amount of triethanolamine during the preparation process were not enough to cause large changes in the Ag distribution on the fabric surface and significant changes in resistivity.

### 3.7. Electromagnetic Shielding

**Figure 8:**
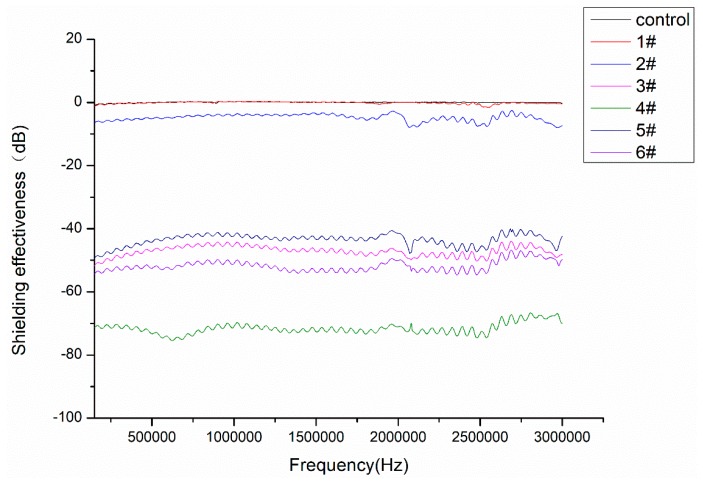
Shielding effectiveness for untreated and treated nonwoven polypropylene.

Figure 8 shows the electromagnetic shielding effectiveness of nonwoven polypropylene before and after Ag coating. It can be observed in Figure 8, the average electromagnetic shielding performance in the control sample was close to 0 dB, while the average value of 4# samples with the best electromagnetic shielding performance reached 71.6 dB, which indicates that this method can be used to prepare polypropylene nonwoven with excellent electromagnetic shielding properties. Compared with the control sample, it can be observed that the electromagnetic shielding effectiveness of 1# sample had not changed significantly, which can be attributed to the fact that silver deposited too little on the surface of the fiber to form a continuous conductive layer on the surface of the fabric and to reflect or absorb electromagnetic waves. Comparing samples 2#, 3#, and 4#, it can be seen that the electromagnetic shielding effectiveness of Ag-coated nonwoven polypropylene increased with the increase of silver nitrate dosage from 0.4 g to 0.8 g in the coating reaction under the same conditions, and the maximum average value reached about 71.6 dB. Similarly, comparing samples 3#, 5#, and 6#, it is found that the electromagnetic shielding effectiveness of Ag coated nonwoven polypropylene increased with the increase of triethanolamine dosage from 20 mL to 60 mL in the coating reaction under the same conditions. These could be attributed to an increase in the amount of silver nitrate or triethanolamine resulting in an increase in the amount of Ag deposited on the surface of the fabric.

## 4. Conclusions

Ag was successfully deposited on nonwoven polypropylene using a simple and convenient technique. SEM and XRD results indicated that silver was uniformly deposited on the surface of the nonwoven polypropylene. The results of FT-IR indicated that Ag deposition on the surface of polypropylene did not change the chemical structure of polypropylene. XPS analysis further confirmed that elemental Ag was deposited on the surface of the nonwoven polypropylene. The TG test results revealed that depositing silver on the surface of the nonwoven polypropylene was beneficial to improve the thermal stability of the polypropylene. The results of the water washing showed that the weight of Ag remaining on the nonwoven after washing for 90 min reached a maximum of 9.62% of the original untreated weight. The surface resistance test results showed that the surface resistivity of the fabric after Ag coating was reduced by more than 60,000 times compared with the original, and the electrical conductivity was improved significantly. After Ag coating, the electromagnetic shielding performance of the fabric was up to 71.6 dB, which was a significant improvement on the electromagnetic shielding effectiveness compare to the nonwoven polypropylene. All the results indicated that this method has great potential in the preparation of Ag functional nonwoven polypropylene and the expansion of their applications.

## Figures and Tables

**Table 1:** Sample preparation conditions.

Sample	Triethanolamine Coating Dosage (mL)	AgNO_3_ Solution	First Boiling (min)	TEA/AgNO_3_ Solution	Second Boiling (min)
Control	0	0	0	0	0
1#	20	0.4 g AgNO_3_ + 200 mLH_2_O	10	0	0
2#	20	0.4 g AgNO_3_ + 200 mLH_2_O	10	0.4 g AgNO_3_ + 40 mL TEA + 170 mL H_2_O	10
3#	20	0.4 g AgNO_3_ + 200 mL H_2_O	10	0.6 g AgNO_3_ + 40 mL TEA + 170 mL H_2_O	10
4#	20	0.4 g AgNO_3_ + 200 mL H_2_O	10	0.8 g AgNO_3_ + 40 mL TEA + 170 mL H_2_O	10
5#	20	0.4 g AgNO_3_ + 200 mL H_2_O	10	0.6 g AgNO_3_ + 20 mL TEA + 170 mL H_2_O	10
6#	20	0.4 g AgNO_3_ + 200 mLH_2_O	10	0.6 g AgNO_3_ + 60 mL TEA + 170 mL H_2_O	10

**Table 2:** Weight change before and after Ag coated polypropylene.

Sample	Untreated Weight (g)	Treated Weight (g)	Weight Gain (g)	Weight Gain Rate (%)
1#	0.9084	0.9882	0.0798	8.78
2#	0.9251	1.117	0.1919	20.74
3#	0.9288	1.176	0.2472	26.61
4#	0.9673	1.2614	0.2941	30.40
5#	0.9291	1.157	0.2279	24.53
6#	0.9095	1.1588	0.2493	27.41

**Table 3:** Weight change for the different washing times.

Sample	Treated Sample Weight (g)	30 min		60 min		90 min	
		Weight (g)	Weight Loss (%)	Weight (g)	Weight Loss (%)	Weight (g)	Weight Loss (%)
1#	0.9882	0.9523	3.63	0.9426	4.61	0.9373	5.15
2#	1.117	1.0177	8.89	0.9974	10.71	0.9873	11.61
3#	1.176	1.0512	10.61	1.0269	12.68	1.0161	13.6
4#	1.2614	1.0968	13.05	1.0653	15.55	1.0533	16.5
5#	1.157	1.0387	10.22	1.0106	12.65	1.001	13.48
6#	1.1588	1.0384	10.39	1.01	12.84	0.9972	13.95

**Table 4:** Surface resistance of untreated and treated nonwoven polypropylene.

Sample	Average Surface Resistance (Ω)	Average Surface Resistivity (Ω)
Control	6.35 × 10^9^	1.26 × 10^10^
1#	5.50 × 10^5^	1.09 × 10^7^
2#	9.20 × 10^3^	1.82 × 10^5^
3#	9.85 × 10^3^	1.95 × 10^5^
4#	9.65 × 10^3^	1.91 × 10^5^
5#	9.44 × 10^3^	1.87 × 10^5^
6#	9.85 × 10^3^	1.95 × 10^5^
